# Sex-related differences in acute coronary syndrome: insights from an observational study in a Yemeni cohort

**DOI:** 10.3389/fcvm.2025.1481917

**Published:** 2025-06-03

**Authors:** Muammar Yahiya Al-Assadi, Nouradden Noman Aljaber, Abdulhafeedh Al-Habeet, Osama Al Nono, Ahmed Al-Motarreb

**Affiliations:** ^1^ Department of Cardiology, Faculty of Medicine, Sana’a university, Sana’a, Yemen; ^2^ Department of Epidemiology and Biostatistics, Faculty of Medical Sciences, Al-Razi University, Sana’a, Yemen

**Keywords:** acute coronary syndrome, sex differences, gender bias, in-hospital mortality, women's health

## Abstract

**Background:**

Acute coronary syndrome (ACS) presents with significant sex-related differences globally, yet research within Yemen remains limited. This study investigates these differences in the clinical presentation, management, and outcomes of Yemeni patients with ACS.

**Methods:**

A retrospective cohort study was conducted at six tertiary care centers, including 1,743 patients (1,379 men and 364 women) hospitalized with ACS between January 2020 and December 2023.

**Results:**

On average, women were generally older than men (59.4 ± 11.7 vs. 57.9 ± 12.7 years, *P* = 0.031) and more frequently diagnosed with non-ST elevation ACS (35.2% vs. 28.9%, *P* = 0.021). Women also exhibited higher rates of traditional cardiovascular risk factors, including diabetes mellitus (31.9% vs. 20.8%, *P* < 0.001) and hypertension (44.5% vs. 32.0%, *P* < 0.001), but had a lower prevalence of atrial fibrillation (0.8% vs. 2.5%, *P* = 0.033) and less likely to engage in ACS lifestyle risk behaviors like smoking (31.0% vs. 83.0%, *P* < 0.001) and khat chewing (53.3% vs. 83.7%, *P* < 0.001). Women were less likely to receive coronary angiography (47.5% vs. 61.3%, *P* < 0.001) or percutaneous coronary intervention (33.8% vs. 46.6%, *P* < 0.001) and were discharged with fewer guideline-recommended therapies for secondary prevention. Women experienced worse in-hospital outcomes, with a significantly higher in-hospital mortality rate (12.6% vs. 7.6%, *P* = 0.002), especially among those with ST-elevation myocardial infarction (STEMI), which remained significant even after adjustment for all clinical confounding factors (adjusted odds ratio, 1.80; 95% CI, 1.16–2.78, *P* = 0.008). However, the one-year mortality rate showed no significant difference between genders.

**Conclusion:**

Yemeni women with ACS experience disparities in treatment and worse in-hospital outcomes, especially in STEMI cases. Addressing gender biases through public health education, healthcare provider training, and infrastructure improvements is essential to improving outcomes.

## 1 Introduction

Cardiovascular diseases (CVDs) are the leading cause of mortality globally, claiming 17.9 million lives in 2019 and constituting 32% of all global deaths. Over three-quarters of these deaths occur in low- and middle-income countries (1). Acute coronary syndrome (ACS), a critical subset of CVDs, includes urgent heart conditions such as myocardial infarction (MI) and unstable angina, necessitating immediate medical intervention (2). Among these, non-ST-segment elevation ACS (NSTE-ACS) encompasses UA and non-ST-segment elevation myocardial infarction (NSTEMI). While both share similar clinical features, NSTEMI is defined by elevated cardiac troponins, reflecting myocardial necrosis, whereas UA presents without biomarker elevation. This distinction carries important prognostic and therapeutic implications, with NSTEMI generally associated with a higher short-term risk and necessitating more aggressive management (3, 4). Several studies have consistently shown sex-related differences in ACS presentations and outcomes. Women, compared to men, were older and more frequently suffered from traditional ACS risk factors and comorbidities, including hypertension (HTN), diabetes mellitus (DM), heart failure (HF), dyslipidemia, and chronic renal failure (CRF) (5–8). Moreover, women often have a lower awareness of ACS symptoms and delay seeking medical care, which can be fatal (6, 8, 9). Alarmingly, women are less likely to receive aggressive pharmacotherapies and invasive treatments, leading to worse outcomes, including higher in-hospital and one-year mortality rates (5, 8, 10, 11).

These disparities are further compounded by sex-specific pathophysiological differences (12). For instance, myocardial infarction with non-obstructive coronary arteries (MINOCA) is more common in women (13, 14), complicating their diagnosis and management. In addition, implicit biases among physicians lead to underestimation of their cardiovascular risk and delayed treatment (15). While younger women (≤45 years) with ACS more often present with typical symptoms, STEMI, and single-vessel disease, and reach the hospital faster than older women (63–64 years), they are less likely to receive guideline-recommended medications. Despite these challenges, younger women demonstrate better in-hospital and 2-year survival rates than older women (16). The present study aimed to investigate the differences in presentation, management, and outcomes between female and male Yemeni patients with ACS. Yemen, an underrepresented population in cardiovascular research, offers a unique perspective, particularly given its distinct socio-economic and healthcare challenges. Most existing ACS studies are conducted in high-income countries, making this study particularly valuable as it sheds light on how sex-related differences manifest in a low-to-middle-income setting. The regional focus of this study contributes a crucial dimension to the existing literature, offering insights into ACS in a population with unique cultural, economic, and healthcare contexts.

## 2 Patients and methods

### 2.1 Study population

We retrospectively evaluated sex-related differences in presentation, management, and outcomes in patients hospitalized with ACS at six tertiary care medical centers in Sana'a City, Yemen (Al Thawra Modern General Hospital, Azal Hospital, Modern European Hospital, University of Science and Technology Hospital, Military Cardiac Center, and Hashim Iraqi Hospital), between January 2020 and December 2023. All patients with ACS aged 18 and older were included, with no exclusion criteria other than missing or incomplete hospital records.

The study was approved by the Research and Ethics Committees of all six participating hospitals and conducted in accordance with the 2013 revision of Declaration of Helsinki. Obtaining informed consent was unnecessary since the data collection was based on a retrospective chart review.

**Figure 1:**
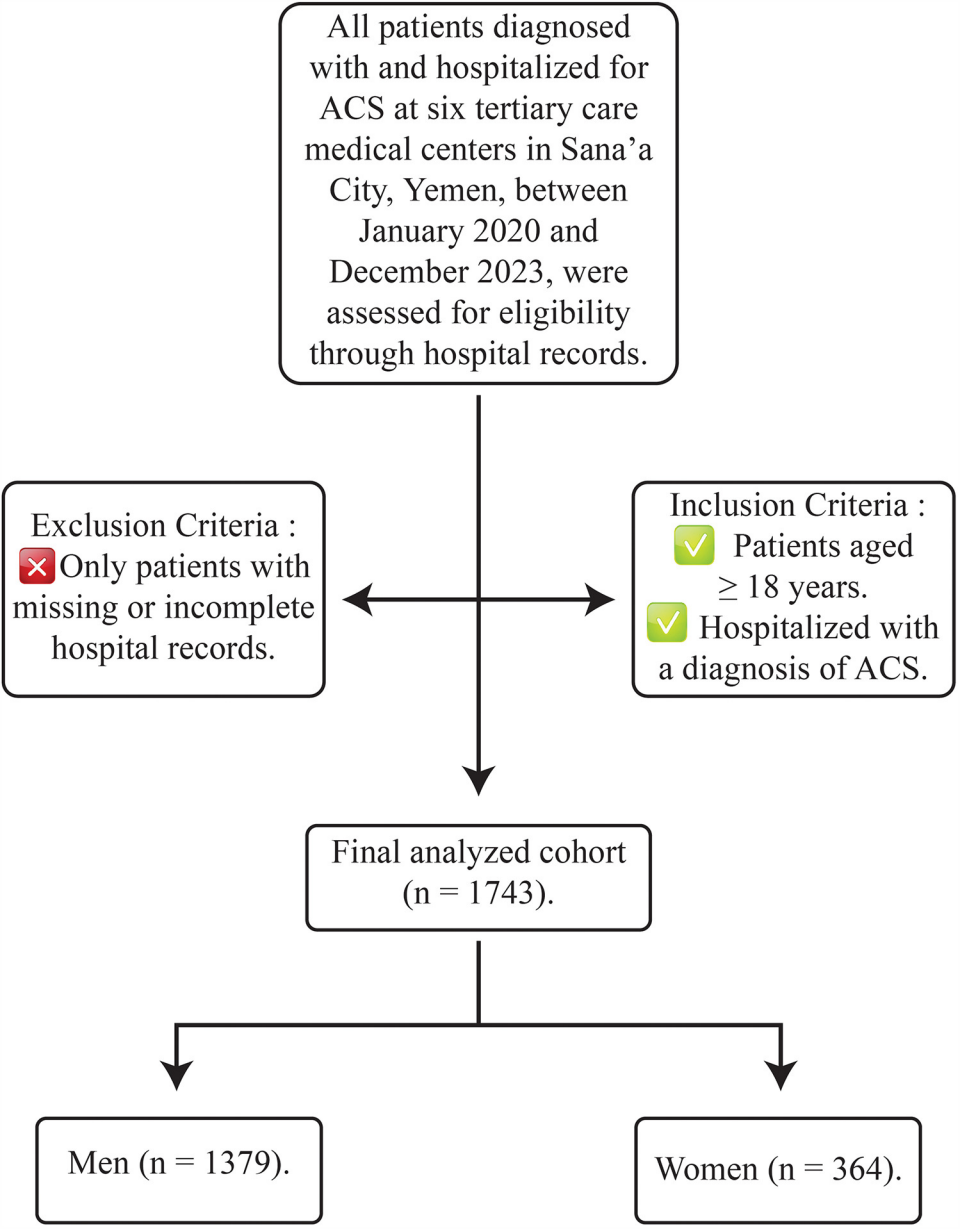
Flowchart of patient selection process.

Figure 1 outlines the inclusion and exclusion criteria applied during the study period, resulting in the final cohort analyzed.

### 2.2 Data collection and study variables

We collected data from various sources, including the statistics department, inpatient care register, catheterization labs register, discharge registry, high-dependency unit register, intensive care unit register, and patient clinical notes. The data was then cross-referenced for accuracy. Demographic and clinical information, such as admission date, age, gender, body mass index (BMI), and coronary risk factors (smoking, HTN, DM, and dyslipidemia) were obtained. Additional details about comorbidities and medical history, including time from symptom onset to admission (in h), chronic renal failure (CRF), atrial fibrillation (AF), valvular heart disease (VHD), cerebrovascular accident (CVA), congestive heart failure (CHF), peripheral arterial disease (PAD), and prior procedures such as percutaneous coronary intervention (PCI) or coronary artery bypass surgery (CABG) were also collected. Also, data on receiving in-hospital and at-discharge guideline-directed medical therapy (GDMT), coronary angiography (CAG), revascularization procedures, and in-hospital adverse outcomes were recorded. All-cause one-year mortality and the corresponding dates of death were verified through follow-up telephone interviews with patients or their next of kin. Clinical data standards set by the American College of Cardiology (ACC) were followed in defining the variables obtained from the patients, adverse in-hospital events, and the diagnosis of ACS types (17).

The diagnosis of acute myocardial infarction (AMI) across all participating centers was established using standardized criteria according to the Fourth Universal Definition of Myocardial Infarction (18), which requires the presence of elevated cardiac troponin levels above the 99th percentile upper reference limit, accompanied by clinical evidence of myocardial ischemia, such as the presence of typical ischemic symptoms, and/or supportive electrocardiogram (ECG) findings.

### 2.3 Statistical analysis

Statistical Package for the Social Sciences (SPSS) version 27 was used for statistical analysis. The Kolmogorov–Smirnov test confirmed that all continuous variables followed a normal distribution, allowing them to be reported as means with standard deviations (SDs). Categorical variables were presented as frequencies and percentages. Comparisons of patient characteristics between women and men were conducted using the Chi-square or Fisher's exact test for categorical variables, and Student's *t*-test for continuous variables. Additionally, the Chi-square was employed to assess the unadjusted risk of in-hospital mortality and 1-year mortality in women compared to men. Multiple binary logistic regression analyses were used to evaluate the adjusted risk of in-hospital mortality separately in patients with NSTEACS and STEMI, adjusting for confounding factors such as age, time from symptom onset to admission, dual antiplatelet therapy (DAPT) at arrival, HTN, DM, and PCI. Odds ratio (OR) and 95% confidence interval (CI) were reported to demonstrate the strength of the association between the risk of female gender and in-hospital mortality. One-year survival was analyzed and represented using Cox regression analysis and Kaplan–Meier curves. Throughout the analysis, two-sided *P*-values less than 0.05 were considered statistically significant.

## 3 Results

### 3.1 Baseline characteristics

A total of 1,743 patients with ACS (1,379 men and 364 women) from six tertiary care centers in Sana'a City, Yemen, were included in the study. Table 1 outlines the baseline characteristics of the participants. On average, women were older than men, with a mean age of 59.4 ± 11.7 years compared to 57.9 ± 12.7 years (*P* = 0.031). Women were more commonly diagnosed with NSTEACS (35.2% vs. 28.9%, *P* = 0.021) and had lower rates of smoking (31.0% vs. 83.0%, *P* < 0.001) and Khat chewing (53.3% vs. 83.7%, *P* < 0.001) compared to men. Additionally, women showed a higher prevalence of traditional cardiovascular risk factors, specifically DM (31.9% vs. 20.8%, *P* < 0.001) and HTN (44.5% vs. 32.0%, *P* < 0.001), while the occurrence of AF was lower in women than in men (0.8% vs. 2.5%, *P* = 0.033).

**Table 1:** Baseline characteristics of the patients.

Baseline characteristics	Overall (*n* = 1,743)	Men (*n* = 1,379)	Women (*n* = 364)	*P*
Age, mean (SD)	58.2 (12.5)	57.9 (12.7)	59.4 (11.7)	**0** **.** **031**
ACS type, *n* (%)				
STEMI	1,216 (69.8)	980 (71.1)	236 (64.8)	**0** **.** **021**
NSTEMI*	527 (30.2)	399 (28.9)	128 (35.2)	
Smoking status, *n* (%)				
Current or ex-smoker	1,258 (72.2)	1,145 (83.0)	113 (31.0)	**<0.001**
Nonsmoker	485 (27.8)	234 (17.0)	251 (69.0)	
Khat chewing status, *n* (%)				
Current or ex-chewer	1,348 (77.3)	1,154 (83.7)	194 (53.3)	**<0.001**
Nonchewer	395 (22.7)	225 (16.3)	170 (46.7)	
Medical history, *n* (%)				
BMI ≥ 30	273 (15.7)	215 (15.6)	306 (84.1)	0.873
Dyslipidemia	230 (13.2)	180 (13.1)	50 (13.7)	0.732
HTN	603 (34.6)	441 (32.0)	162 (44.5)	**<0.001**
DM	403 (23.1)	287 (20.8)	116 (31.9)	**<0.001**
CRF	9 (0.5)	7 (0.5)	2 (0.5)	0.591
VHD	15 (0.9)	12 (0.9)	3 (0.8)	0.615
AF	37 (2.1)	34 (2.5)	3 (0.8)	**0** **.** **033**
Angina	500 (28.7)	390 (28.3)	110 (30.2)	0.467
MI	263 (15.1)	213 (15.4)	50 (13.7)	0.418
CCF	69 (4.0)	56 (4.1)	13 (3.6)	0.670
CVA	71 (4.1)	53 (3.8)	18 (4.9)	0.344
PAD	32 (1.8)	21 (1.5)	11 (3.0)	0.058
Time from symptom onset to admission (h), mean (SD)	12.6 (6.4)	12.4 (6.5)	13.2 (5.8)	0.071

AF, atrial fibrillation; BMI, body mass index; CCF, congestive cardiac failure; CRF, chronic renal failure; CVA, cerebral vascular accident; DM, diabetes mellitus; HTN, hypertension; MI, myocardial infarction; NSTEMI, Non-ST-elevation myocardial infarction; PAD, peripheral artery disease; STEMI, ST-elevation myocardial infarction; VHD, valvular heart disease. Note: Values presented in bold denote statistically significant associations (*P* < 0.05).

* Unstable angina was diagnosed in 54.2% (286/528) of the subgroup, showing significantly higher prevalence in women (64.8%, 83/128) than men (50.8%, 203/399; *P* = 0.002).

### 3.2 Guideline-directed medical therapy and procedures

Table 2 shows the use of GDMT and procedures across different ACS types. The study population showed a high use of DAPT upon arrival, as well as in-hospital ACEIs/ARBs (angiotensin-converting enzyme inhibitors/angiotensin receptor blockers) and statins, with no significant differences between women and men, even after subgroup analysis by ACS type. Although the overall use of beta-blockers (BB) was satisfactory, it was lower in women than men (57.4% vs. 64.9%, *P* = 0.008); however, this difference did not persist in the subgroup analysis by ACS type. Among the 1,743 ACS patients, 1,019 (58.5%) underwent CAG within 24 h of admission. Women were less likely than men to receive this procedure (47.5% vs. 61.3%, *P* < 0.001), and this disparity remained significant after the subgroup analysis by ACS type. Similarly, the rate of PCI procedures was significantly lower in women compared to men (33.8% vs. 46.6%, *P* < 0.001) across all ACS types.

**Table 2:** Guideline-directed medical therapy and procedures by ACS type.

Therapy/procedure	Overall (*n* = 1,743)	Men (*n* = 1,379)	Women (*n* = 364)	*P*
In-hospital GDMT and procedures				
All ACS, *n* (%)				
DAPT at arrival	1,533 (88.0)	1,212 (87.9)	321 (88.2)	0.877
BB	1,104 (63.3)	895 (64.9)	209 (57.4)	**0** **.** **008**
ACEIs/ARBs	1,332 (76.4)	1,053 (76.4)	279 (76.6)	0.908
Statins	1,630 (93.5)	1,293 (93.8)	337 (92.6)	0.416
CAG	1,019 (58.5)	846 (61.3)	173 (47.5)	**<0.001**
PCI	766 (43.9)	643 (46.6)	123 (33.8)	**<0.001**
STEMI, *n* (%)				
DAPT at arrival	1,079 (88.7)	867 (88.5)	212 (89.8)	0.553
BB	790 (65.0)	649 (66.2)	141 (59.7)	0.061
ACEIs/ARBs	917 (75.4)	732 (74.7)	185 (78.4)	0.237
Statins	1,142 (93.9)	925 (94.4)	217 (91.9)	0.159
CAG	778 (64.0)	647 (66.0)	131 (55.5)	**0** **.** **003**
PCI	645 (53.0)	537 (54.8)	108 (45.8)	**0** **.** **013**
NSTEACS, *n* (%)				
DAPT at arrival	454 (86.1)	345 (86.5)	109 (85.2)	0.709
BB	314 (59.6)	246 (61.7)	68 (53.1)	0.087
ACEIs/ARBs	415 (78.7)	321 (80.5)	94 (73.4)	0.092
Statins	488 (92.6)	368 (92.2)	120 (93.8)	0.568
CAG	241 (45.7)	199 (49.9)	42 (32.8)	**0** **.** **001**
PCI	121 (23.0)	106 (26.6)	15 (11.7)	**0** **.** **001**
GDMT for secondary prevention at discharge in all ACS patients, *n* (%)				
DAPT	1,410 (80.9)	1,126 (81.7)	284 (78.0)	0.117
BB	1,170 (67.1)	943 (68.4)	227 (62.4)	**0** **.** **030**
ACEIs/ARBs	1,410 (80.9)	1,127 (81.7)	283 (77.7)	0.086
Statins	1,522 (87.3)	1,218 (88.3)	304 (83.5)	**0** **.** **014**

ACEIs/ARBs, angiotensin-converting enzyme inhibitors/angiotensin receptor blockers; BB, beta blocker; CAG, coronary angiography; DAPT, dual antiplatelet therapy; GDMT, guideline-directed medical therapy; PCI, percutaneous coronary intervention. Note: Values presented in bold denote statistically significant associations (*P* < 0.05).

### 3.3 Gender-related differences in adverse in-hospital outcomes by ACS type

Table 3 presents a comparison of adverse in-hospital outcomes between men and women based on ACS type. Women exhibited higher rates of adverse outcomes compared to men, including CHF (24.5% vs. 18.3%, *P* = 0.009), recurrent ischemia (35.4% vs. 26.1%, *P* < 0.001), and mortality (12.6% vs. 7.6%, *P* = 0.002). These differences were particularly pronounced in patients with STEMI. In contrast, for patients with NSTEACS, only recurrent ischemia remained significantly higher in women than in men (32.8% vs. 22.8%, *P* = 0.023).

**Table 3:** Gender-related differences in in-hospital outcomes by ACS type.

In-hospital outcome	Overall (*n* = 1,743), *n* (%)	Men (*n* = 1,379), *n* (%)	Women (*n* = 364), *n* (%)	*P*
All ACS, *n* (%)				
Major bleeding	12 (0.7)	9 (0.7)	3 (0.8)	0.473
Strock	19 (1.1)	17 (1.2)	2 (0.5)	0.208
Recurrent ischemia	489 (28.1)	360 (26.1)	129 (35.4)	**<0.001**
Shock	216 (12.4)	163 (11.8)	53 (14.6)	0.158
Re-infraction	70 (4.0)	55 (4.0)	15 (4.1)	0.909
CCF	342 (19.6)	253 (18.3)	89 (24.5)	**0** **.** **009**
Mortality	151 (8.7)	105 (7.6)	46 (12.6)	**0** **.** **002**
STEMI, *n* (%)				
Major bleeding	6 (0.5)	6 (0.6)	0 (0.0)	0.273
Strock	13 (1.1)	13 (1.3)	0 (0.0)	0.060
Recurrent ischemia	356 (29.3)	269 (27.4)	87 (36.9)	**0** **.** **004**
Shock	160 (13.2)	122 (12.4)	38 (16.1)	0.136
Re-infraction	64 (5.3)	51 (5.2)	13 (5.5)	0.851
CCF	240 (19.7)	182 (18.6)	58 (24.6)	**0** **.** **037**
Mortality	122 (10.0)	86 (8.8)	36 (15.3)	**0** **.** **003**
NSTEACS, *n* (%)				
Major bleeding	6 (1.1)	3 (0.3)	3 (2.3)	0.140
Strock	6 (1.1)	4 (1.0)	2 (1.6)	0.450
Recurrent ischemia	133 (25.2)	91 (22.8)	42 (32.8)	**0** **.** **023**
Shock	56 (10.6)	41 (10.3)	15 (11.7)	0.645
Re-infraction	6 (1.1)	4 (1.0)	2 (1.6)	0.603
CCF	102 (19.4)	71 (17.8)	31 (24.2)	0.109
Mortality	29 (5.5)	19 (4.8)	10 (7.8)	0.188

CCF indicates congestive cardiac failure. Note: Values presented in bold denote statistically significant associations (*P* < 0.05).

### 3.4 Univariate and multivariate analyses of gender-related differences in in-hospital mortality by ACS type

Univariate and multivariate analyses were performed separately on patients with NSTEACS and STEMI and presented in Table 4. Among the NSTEACS patients, there was no significant difference in in-hospital mortality between men and women, even after adjusting for all clinical factors (AOR, 2.87, 95% CI, 0.91–5.18, *P* = 0.081). However, in the STEMI patient group, women were nearly two times more likely to in-hospital mortality (crude odd ratio, 1.87; 95% CI, 1.23–2.84, *P* = 0.003). This increased risk remained significant even after adjusting for age (AOR, 1.80; 95% CI, 1.18–2.75; *P* = 0.006), age and comorbidities (AOR, 1.80; 95% CI, 1.18–2.75; *P* = 0.006), and age, comorbidities, and the time from symptom onset to admission (AOR, 1.73; 95% CI, 1.12–2.67; *P* = 0.012). Further adjustments were made for using DAPT upon arrival and PCI to determine if these factors could explain the remaining sex differences in in-hospital mortality. Women still had a higher in-hospital mortality risk even after further adjusting for DAPT use at arrival (AOR, 1.76; 95% CI, 1.14–2.71; *P* = 0.011) and PCI (AOR, 1.80; 95% CI, 1.16–2.78; *P* = 0.008).

**Table 4:** Univariate and multivariate analyses of gender-related differences in in-hospital mortality by ACS type.

Model	STEMI		NSTEACS	
	OR (95% CI)	*P*	OR (95% CI)	*P*
In-hospital mortality				
Unadjusted	1.87 (1.23–2.84)	**0** **.** **003**	1.69 (0.76–3.74)	0.192
Model 1^a^	1.80 (1.18–2.75)	**0** **.** **006**	1.61 (0.72–3.62)	0.243
Model 2^b^	1.75 (1.14–2.70)	**0** **.** **010**	1.60 (0.71–3.63)	0.253
Model 3^c^	1.73 (1.12–2.67)	**0** **.** **012**	1.68 (0.74–3.83)	0.213
Model 4^d^	1.76 (1.14–2.71)	**0** **.** **011**	1.66 (0.72–3.82)	0.229
Model 5^e^	1.80 (1.16–2.78)	**0** **.** **008**	2.19 (0.91–5.25)	0.078

CI, confidence interval; NSTE-ACS, non–ST-segment–elevation acute coronary syndrome; OR, odds ratio; STEMI, ST-segment–elevation myocardial infarction.

^a^ Model 1 was adjusted for age.

^b^ Model 2 was adjusted for age and significant comorbidities in Table 1 (HTN, DM, and AF).

^c^ Model 3 was adjusted for model 2 plus time from symptom onset to admission.

^d^ Model 4 was adjusted for model 3 plus DAPT at arrival.

^e^ Model 5 was adjusted for model 4 plus PCI.

Notes: Men were the reference group throughout the analysis of all models. Values presented in bold denote statistically significant associations (*P* < 0.05).

### 3.5 Gender-related differences in the all-cause one-year mortality rate

**Figure 2:**
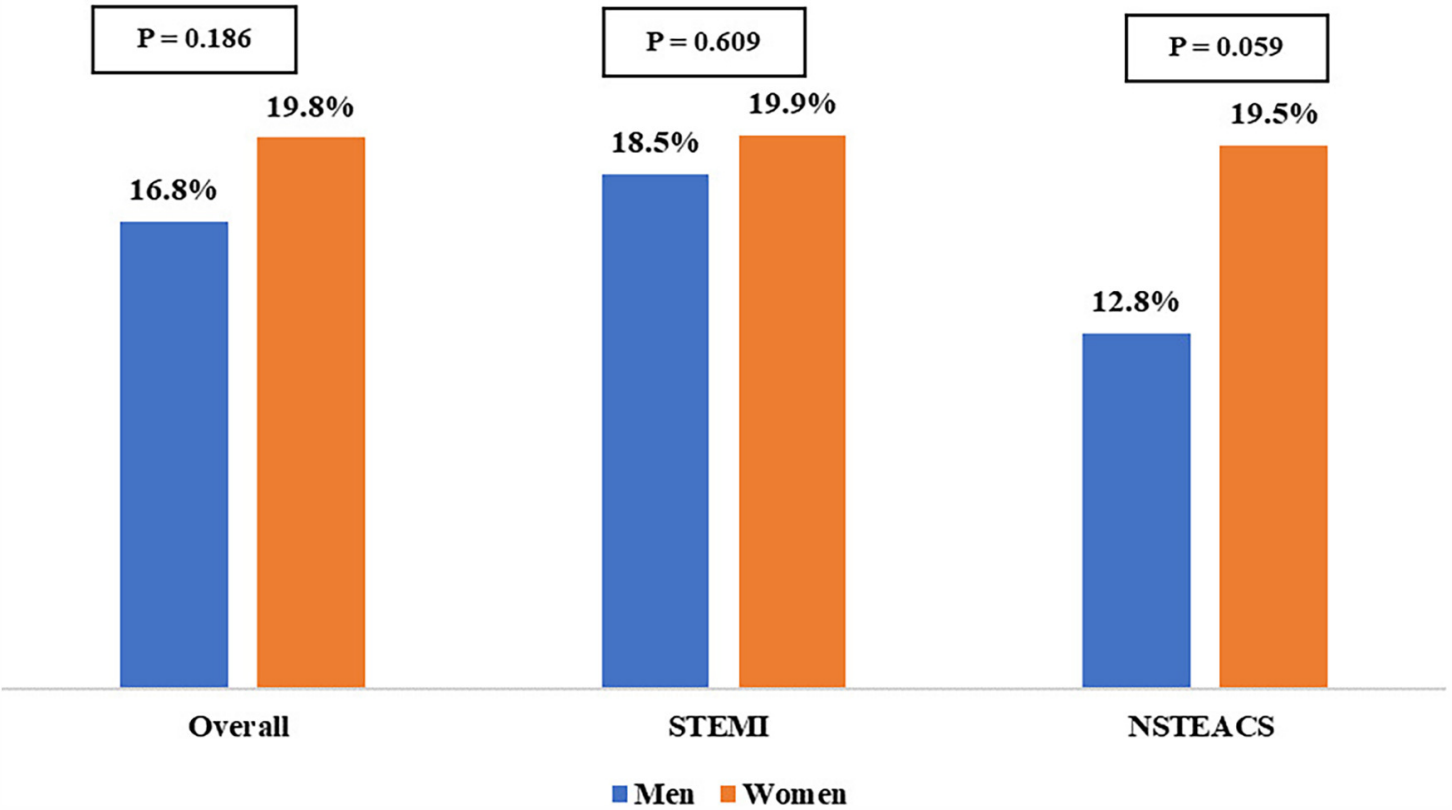
Gender-related differences in all-cause one-year mortality by ACS type. NSTE-ACS, non–ST-segment–elevation acute coronary syndrome; STEMI, ST-segment–elevation myocardial infarction.

**Figure 3:**
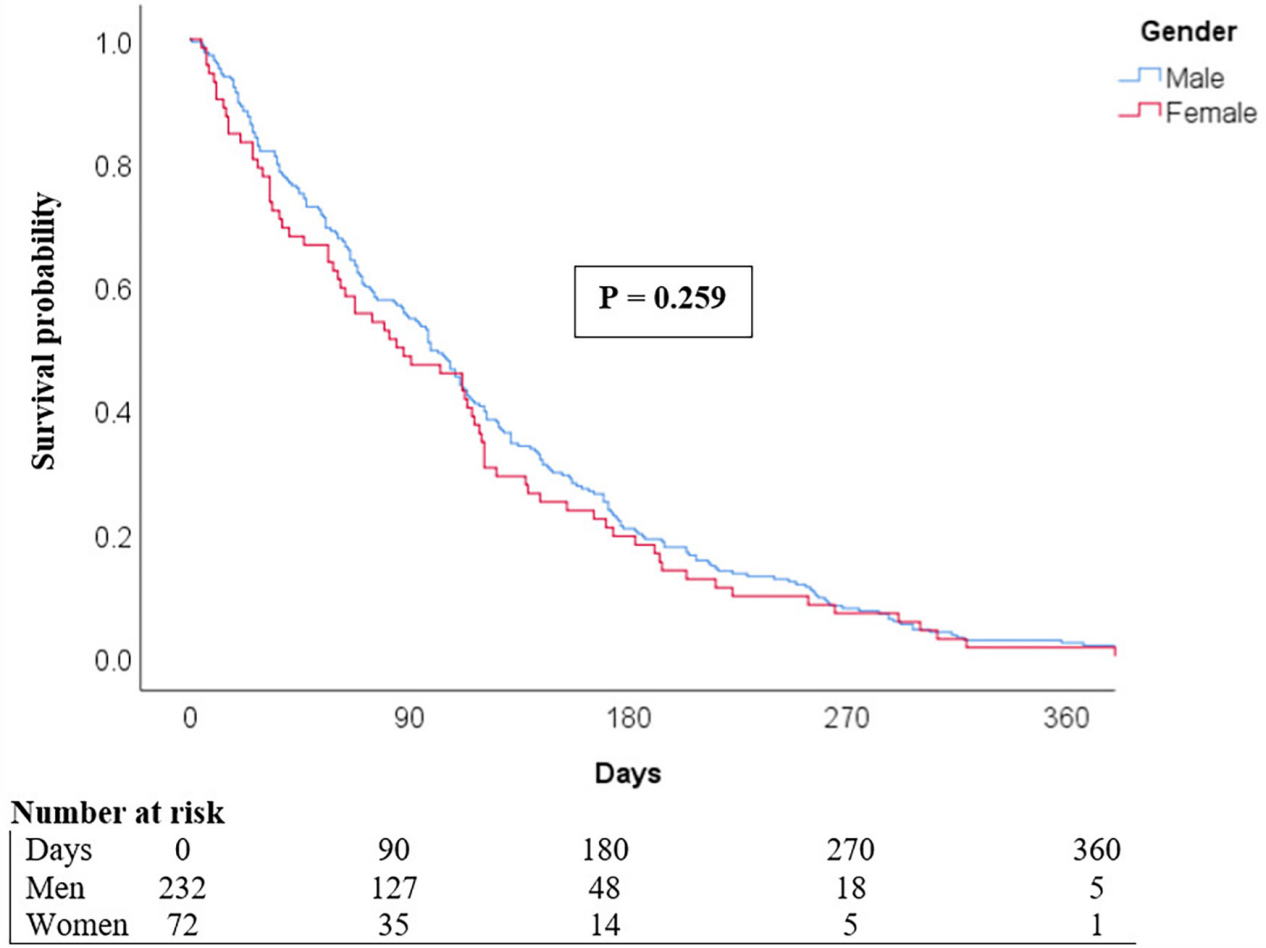
Kaplan–Meier curves compare the survival of men and women with ACS over the year following hospital admission.

As shown in Figure 2, all-cause one-year mortality did not significantly differ between women and men, regardless of ACS type. Similarly, the Kaplan–Meier curve showed no significant difference in one-year survival between female and male patients in the ACS population (*P* = 0.259) (Figure 3).

## 4 Discussion

To our knowledge, this multicenter study is the first of its kind to investigate sex-related differences in the presentation, management, and outcomes of Yemeni patients with ACS. It is also one of the largest studies in Middle Eastern countries to date. The study found that women were generally older and more likely to be diagnosed with NSTEACS compared to men. Additionally, women presented more with traditional cardiovascular risk factors such as HTN and DM. Still, they had a lower prevalence of AF and were less likely to engage in ACS lifestyle risk behaviors like smoking and khat chewing. Among the key findings was that women were less likely than men to undergo CAG and PCI. Additionally, they received fewer GDMT for secondary prevention at discharge, such as BB and statins. Nevertheless, women were more likely to have worse in-hospital outcomes, including CCF, recurrent ischemia, and mortality, especially in patients with STEMI, which persisted after adjustment for clinical factors. Due to its contemporary data and relatively large sample size, our study adds to the existing evidence on sex-related differences in ACS patients. This is particularly relevant given the underrepresentation of Middle Eastern populations in cardiovascular research.

The complexities of sex differences in ACS patients stem from both biological factors and societal biases (19). Our data reveals a women-to-men ratio of 1:4, aligning with findings from the Malaysian National Cardiovascular Disease Database and a tertiary hospital in Kenya (5, 20). Historically, men have exhibited higher ACS rates. Still, this disparity is diminishing, particularly in developed nations, underscoring the increasing awareness of ACS as a significant health concern among younger women (21). Recent data from Germany further illustrates this trend, showing a women-to-men ratio of 1.9:1 (10).

In line with documented epidemiological data (5, 6, 11), our data indicated that women are more susceptible to traditional ACS risk factors, such as DM and HTN, whereas men are more likely to engage in ACS lifestyle risk behaviors like smoking. A recent study emphasizes that these risk factors, mainly DM, HTN, and hypercholesterolemia, are strongly linked to ACS in young women (22). The VIRGO study further highlights that the impact of individual risk factors, such as DM, obesity, and smoking, is more pronounced in women (23). For instance, DM increases the risk of MI fourfold in women, compared to 2.5 times in men (24). While estrogen offers some protection against ACS in young women, this effect may diminish in those with DM (25).

Recent advancements in high-sensitivity troponin testing have significantly improved early detection of ACS, particularly STEMI, yet a prevalent misconception among healthcare providers and the community is that women have a low risk of this condition (26). This belief hampers primary prevention efforts, resulting in women receiving inadequate preventative therapies and lifestyle counseling and delaying timely presentation, which can be fatal, especially in AMI (19). Although statistically unproven, our data indicates that women have a longer delay seeking hospital care than men (13.2 h vs. 12.4 h, *P* = 0.071). This finding is likely due to unawareness of ACS symptoms in women, as well as societal norms in Yemen that limit women's healthcare access. Women often experience atypical symptoms, delaying ACS recognition (27, 28); however, recent literature reveals significant symptom overlap between sexes (29, 30), with around 90% of both men and women experiencing chest pain or pressure as the primary symptom (31, 32). Women typically report additional symptoms alongside chest pain (31, 32), prompting the American College of Cardiology/American Heart Association to recommend avoiding the term “atypical” for chest pain, as it can lead to underdiagnosis and less intensive care for women, exacerbating sex and gender-related disparities in ACS treatment (33).

Despite the benefits of invasive procedures and GDMT for both sexes, women with ACS are less likely to receive GDMT, timely reperfusion therapies, and revascularization compared to men. Our findings indicate that women are less likely to undergo CAG and receive reperfusion therapy with primary PCI than men, a trend supported by other studies, especially in STEMI patients (5, 10, 11). This discrepancy may be partly due to the longer delay between symptom onset and hospital admission for women. Although there is a closing gap in improvement in cardiac care between men and women, differences in treatment strategies continue to exist, particularly pertaining to statin therapy at discharge (34). Our findings show that women are less likely to receive GDMT like statins and BB, at discharge, which can impact long-term outcomes and perpetuate gender-related disparities in cardiac care. One study suggested that disparities in medical insurance coverage contribute to this gap, but even with adjustments for insurance status, women were still less likely to receive GDMT (11).

In Yemen and other low-income settings, limited healthcare resources and minimal insurance coverage pose significant challenges, particularly for women with less financial autonomy. Our data shows that women experience worse in-hospital outcomes, including CCF, recurrent ischemia, and mortality, especially in STEMI patients, which persisted statistically significantly even after adjustment for clinical factors. Data from China and the USA corroborate this finding, showing that women face a higher risk of in-hospital mortality in STEMI cases, often due to delayed recognition of ACS and insufficient symptom awareness (11, 35). Research involving data from over 1,000 hospitals also revealed higher in-hospital mortality rates in younger women compared to men, often due to the absence of chest pain (21). Recently, it has been suggested that high-quality PCI centers can reduce sex differences in in-hospital mortality (36); however, the ongoing disparity in care emphasizes the need for focused interventions. Efforts like the Chinese National Chest Pain Centers Program have reduced gender-related disparities by coordinating prehospital and in-hospital treatment and standardizing procedures (37). In Yemen, limited resources, especially in rural areas, hamper the implementation of a robust ACS care system. Recent evidence indicates lower early mortality rates in ACS patients when the door-to-balloon time is ≤90 min (36). While PCI usage has recently increased in Yemen, poor road conditions and a lack of reliable transportation can delay the transfer of patients to facilities capable of providing PCI within the critical window. A more practical approach might be the “hub and spoke” model from Tamil Nadu, India, involving initial treatment at smaller hospitals with transfers to more extensive facilities for further care (38). Nevertheless, this model still requires substantial resources and coordination. Therefore, immediate priorities include raising STEMI symptom awareness, improving emergency medical services, ensuring thrombolytic availability, and educating physicians on timely reperfusion. Ultimately, substantial investment in Yemen's healthcare infrastructure is essential to establish an effective ACS care system.

Although women in our cohort underwent significantly fewer revascularization procedures, the numerically higher one-year all-cause mortality observed in women (19.8%) compared to men (16.8%) did not reach statistical significance (*P* = 0.186). Several factors could explain the absence of statistical significance in this situation. First, the use of all-cause mortality (rather than cardiovascular-specific mortality) may have obscured sex differences in cardiac-related deaths. Second, women had a significantly lower prevalence of AF and less exposure to harmful behavioral risk factors (e.g., smoking), which could have attenuated one-year mortality disparities. Third, unmeasured confounders, such as medication adherence, could have influenced one-year all-cause mortality beyond acute-phase treatments. Additionally, women who survived initial hospitalization despite worse in-hospital outcomes might represent a selected subgroup with more favorable prognostic features, introducing a potential survivor bias. Crucially, the lack of coronary angiographic data limited our ability to identify or characterize MINOCA, a condition with well-documented sex-specific differences (39, 40). These limitations underscore the need for future studies incorporating angiographic analysis, systematic documentation, and comprehensive risk stratification to better elucidate sex-based disparities in ACS outcomes.

### 4.1 Limitations

The present study has several limitations. Firstly, its retrospective design poses an inherent selection bias, as the reliability of such analyses is contingent on the quality of the documentation system. As such, the study population was restricted to hospitalized patients with ACS, thus excluding patients who died before reaching the hospital, which could affect the generalizability of the findings. Secondly, certain clinical data were not consistently available across all participating centers. In particular, the lack of standardized angiographic documentation hindered the reliable identification of MINOCA, while the absence of variables required for validated risk scores (such as TIMI or GRACE) precluded proper risk stratification in patients with NSTIMI. Thirdly, the clinical profiles of women were generally worse compared to men. Although these differences were adjusted for in the regression analysis, residual confounding from both measured and unmeasured factors may still exist. Specifically, the higher incidence of AF in men, potentially linked to elevated cardiac troponin and ECG abnormalities, could have influenced treatment decisions such as DAPT and PCI, contributing to observed sex disparities. On the positive side, the study population was relatively large and contemporary, and key clinical data were available for analysis, enabling us to tighten control for potential confounding factors. The study also provides a foundation for future prospective investigations into sex-related disparities in ACS management and outcomes in Yemen.

## 5 Conclusions

This multicenter study, one of the largest in Middle Eastern countries and the first of its kind in Yemen, offered valuable insights into gender-related differences in patients admitted with ACS. The findings indicated that Yemeni women hospitalized with ACS are less likely to undergo invasive procedures or receive secondary prevention strategies, leading to worse in-hospital outcomes, particularly in STEMI cases. Nevertheless, the underlying reasons behind these gender-related differences in clinical management remain unclear. To address these disparities, it is essential to tackle gender biases in healthcare through public health education, improved training for healthcare providers, and significant enhancements to healthcare infrastructure. Further prospective research into gender-related differences in ACS management and outcomes is urgently needed.

## Data Availability

The original contributions presented in the study are included in the article/Supplementary Material, further inquiries can be directed to the corresponding author.
